# Case report expanding the germline *AXIN2-* related phenotype to include olfactory neuroblastoma and gastric adenoma

**DOI:** 10.1186/s12881-020-01103-0

**Published:** 2020-08-17

**Authors:** Sarah K. Macklin- Mantia, Stephanie L. Hines, Kaisorn L. Chaichana, Angela M. Donaldson, Stephen L. Ko, Qihui Zhai, Niloy Jewel Samadder, Douglas L. Riegert-Johnson

**Affiliations:** 1 Department of Clinical Genomics, Mayo Clinic, 4500 San Pablo Road South, Jacksonville, FL 32224 USA; 2 Department of Medicine, Division of Diagnostic & Consultative Medicine, Mayo Clinic, 4500 San Pablo Road South, Jacksonville, FL 32224 USA; 3 Department of Neurologic Surgery, Mayo Clinic, 4500 San Pablo Road South, Jacksonville, FL 32224 USA; 4 Department of Otolaryngology, Mayo Clinic, 4500 San Pablo Road South, Jacksonville, FL 32224 USA; 5 Department of Radiation Oncology, Mayo Clinic, 4500 San Pablo Road South, Jacksonville, FL 32224 USA; 6 Department of Laboratory Medicine and Pathology, Mayo Clinic, 4500 San Pablo Road South, Jacksonville, FL 32224 USA; 7 Department of Gastroenterology, Mayo Clinic, 5777 E. Mayo Boulevard, Phoenix, AZ 85054 USA; 8 Department of Gastroenterology, Mayo Clinic, 4500 San Pablo Road South, Jacksonville, FL 32224 USA

**Keywords:** *AXIN2*, Hereditary cancer syndrome, Hereditary polyposis, Hereditary colorectal cancer, Hypodontia, Olfactory neuroblastoma, Gastric adenomas

## Abstract

**Background:**

Pathogenic *AXIN2* variants cause absence of permanent teeth (hypodontia), sparse hair and eye brows (ectodermal dysplasia), and gastrointestinal polyps and cancer. Inheritance is autosomal dominant with variable penetrance. Only twenty- five patients have been reported from five families. A Mayo Clinic pilot program tested 3009 newly diagnosed cancer patients for pathogenic germline variants in 83 hereditary cancer genes, including *AXIN2*. We found only one patient with a pathogenic *AXIN2* variant.

**Case presentation:**

The proband was a 49 year-old female who came to Otolaryngology clinic complaining of right-sided nasal obstruction. Biopsy of identified nasal polyp revealed olfactory neuroblastoma (esthesioneuroblastoma). Surgical resection with gross, total tumor resection was followed by radiation therapy. The patient enrolled in a clinical pilot of genetic testing and a pathogenic variant in *AXIN2*, c.1822del (p.Leu608Phefs*81) (NM_004655.3) was found. She was seen in Medical Genetics clinic and found to have a personal history of hypodontia. Her eyebrows, hair, and nails were all normal. She underwent upper endoscopy and colonoscopy. A four mm gastric adenoma was found and removed.

**Conclusions:**

This is the first case reported on a patient with a pathogenic, germline *AXIN2* variant and an olfactory neuroblastoma or a gastric adenoma. We propose that these could be features of the *AXIN2* phenotype. The known association between gastric adenomas and familial adenomatous polyposis, the other Wnt/beta-catenin disorder, supports the hypothesis that pathogenic *AXIN2* variants increase risk as well. As the odds of a chance co-occurrence of a pathogenic *AXIN2* variant and an olfactory neuroblastoma are so rare, it is worth exploring potential causation. We are building a clinical registry to expand understanding of the AXIN2 phenotype and request any clinicians caring for patients with pathogenic AXIN2 variants to contact us.

## Background

AXIN2, AXIN1, APC, and GSK-3 beta comprise the beta-catenin destruction complex of the canonical Wnt signaling pathway (MIM604025). Somatic pathogenic variants in *APC* and the other members of the beta-catenin destruction complex occur in most colorectal cancers and in many other cancers. Germline pathogenic variants of the beta catenin destruction complex genes have only been reported in *AXIN2* and *APC*.

*AXIN2* pathogenic variants are associated with the absence of permanent teeth (hypodontia), sparse hair and eye brows (ectodermal dysplasia), and gastrointestinal (GI) polyps and cancer. Inheritance is autosomal dominant with variable penetrance. Four of five pathogenic *AXIN2* variants reported result in a frameshift with premature termination (Trp663*, Arg656*, Asn666*, Ser658*,); one is a missense substitution (Arg463Cys) [1–4].

*AXIN2* pathogenic variants are rare. Only 25 patients have been reported in five families [1–4]. The first family was published 17 years ago in 2004 [1]. There were 11 individuals in a 4 generation Finnish pedigree and an additional de novo case included. All cases had absence of teeth, with 11 of 12 missing 8 or more teeth. Nine of the patients had had colonoscopy and the findings ranged from normal (1) to hyperplastic polyps and adenomas without dysplasia (5), adenomas with severe dysplasia (2), and adenocarcinoma (1).

In the second family, there were 4 confirmed *AXIN2* pathogenic variant carriers: three sisters and the daughter of one sister [2]. All four had oligodontia. All three of the sisters had colorectal neoplasia. One had polyposis (> 100 adenomas) with two surgeries resulting in a subtotal colectomy. Another had metachronous colon cancers at 50 and 59 years of age and breast cancer at age 44. The last sister had a history of colon polyps, without further details available. The daughter had completed colonoscopy and an upper endoscopy at 34 years of age. She had cystic fundic gland stomach polyps. This is the only *AXIN2* family with ectodermal dysplasia. Two family members had absent eyebrows and sparse hair, and another had sparse eyebrows. The mother of the three sisters had oligodontia, absent eyebrows, and sparse hair, with no known history of malignancy. She died at 97 years of age and never had genetic testing.

The third family was Spanish family of four; a father, two daughters, and a son [3]. Three of the four had been diagnosed with colon cancer at ages 36, 42, and 51 years of age. One of the daughters completed colonoscopy at age 43, which was negative. None of the family members had oligodontia or other features of ectodermal dysplasia.

The most recent family was an Australian mother and her three children [4]. The mother was the proband. She came to a medical genetics clinic for her history of over 100 adenomatous polyps. After testing positive for pathogenic AXIN2 variant she was discovered to have history of oligodontia. Her daughter and two sons both all had oligodontia and colonic polyposis. The daughter was diagnosed with colon cancer at 43 years of age and died at 46.

We report the 26 patient with a novel *AXIN2* pathogenic germline variant. Given the rarity of the disorder, each additional case report is useful in defining the natural history and informing patient care. This is the first *AXIN2* germline pathogenic variant case reported with an olfactory neuroblastoma and also the first case with a gastric adenoma.

Outside of a medical genetics clinic, it is very unlikely the *AXIN2* phenotype would be recognized by a clinician. This family was identified through a Mayo Clinic pilot program testing patients newly diagnosed with all types of cancer for germline variants in 83 cancer- related genes, including *AXIN2* (INTERCEPT study). In 2018 and 2019, 3009 patients were tested; only one pathogenic *AXIN2* germline variant was found.

## Case presentation

The proband was a 49 year-old female. She was referred to the Mayo Clinic for evaluation and treatment of a right-sided nasal mass. At initial consultation in the Otolaryngology clinic, the patient complained of a 3 month history of right-sided nasal symptoms, including progressive congestion, decreased sense of smell, and epistaxis. She also reported mild right posterior orbital pain with slight right eye swelling, occipital pain, upper gum pain, and progressive decrease in sense of taste.

**Fig. 1:**
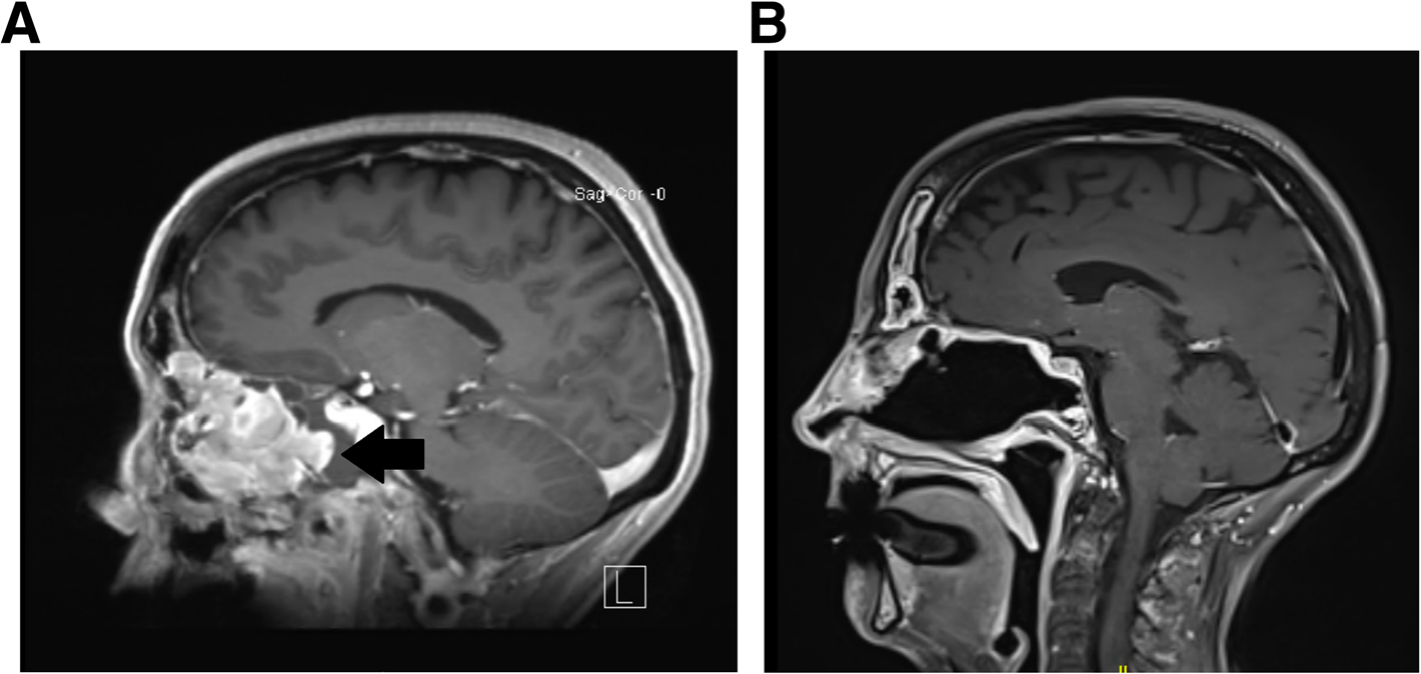
MRI Imaging of the Olfactory Neuroblastoma. Pre (**a**) and post (**b**) operative sagittal brain MRIs from our proband. The arrow on the left image indicates the olfactory neuroblastoma (neuroesthioblastoma)

The patient had a normal physical exam, except for unilateral cervical lymphadenopathy. On nasal endoscopy a grade 3 polyp was noted in the middle meatus. An intranasal biopsy was performed identifying cells consistent with a neuroendocrine malignancy, most likely olfactory neuroblastoma (esthesioneuroblastoma). Subsequent magnetic resonance imaging (MRI), showed a tumor in her right nasal cavity with thickening of the right frontal sinus, ethmoids, and sphenoid sinus. There was contact with the dura, as well as, medial orbital wall and maxillary sinus [Fig. 1a]. Given the tumor’s proximity to the skull base a neurosurgery consultation was obtained.

The patient was brought to the operating room about 3 months after she first sought medical attention, and 6 months after her symptoms began. The patient had a purely endoscopic resection of the tumor, which included bilateral maxillary antrostomies, total ethmoidectomies, frontal sinus Draf III procedure, and dural biopsy with skull base reconstruction using a left nasoseptal flap. The total operative time was 300 min, and the patient was discharged home on post-operative day 2 with no complications.

**Fig. 2:**
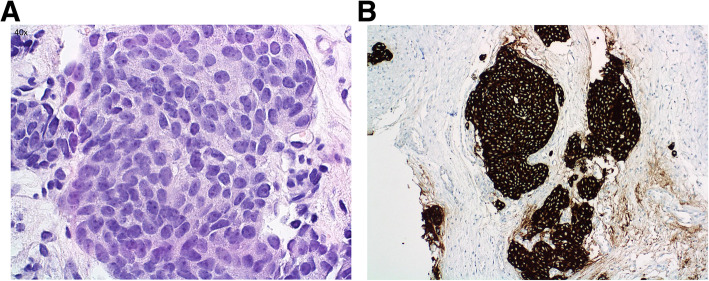
Pathology from Olfactory Neuroblastoma. Histopathological images from the olfactory neuroblastoma (esthesioneuroblastoma) removed from our proband at surgery. Images shown are from the tumor tissue resected from the right frontal nasal sinus. Panel A shows a nest of tumor cells with the characteristic “salt and pepper” appearance (hematoxylin and eosin staining 40x). Panel B shows intense immunohistochemical staining for synaptophysin supporting the diagnosis of neuroblastoma (20x). Several other immunostains were done but not shown. Tumor cells were also positive for chromogranin, and negative for CAM 5.2, EMA, and CD45; GFAP is essentially negative in the matrix

The operative pathology report confirmed the diagnosis of olfactory neuroblastoma, Hyams grade 3 and Kadish stage C [Fig. 2]. The pathologist confirmed tumor in the Vidian canal, skull base, right sphenoid and right frontal sinus. No definitive tumor was identified in the right maxillary margin, but the impression was that the margin was close. MRI the day following surgery showed gross total resection of the tumor [Fig. 1b].

Thirty days after surgery, the patient began radiation. She was treated to a dose of 60 Gy in 30 fractions. At the time this paper was submitted, the patient was over 390 days post-surgery. The patient’s most recent imaging was 363 days after surgery and showed no recurrence.

Germline genetic testing was ordered through a commercial genetic testing company and included analysis of 83 genes*.* A pathogenic variant was detected in *AXIN2,* c.1822del (p.Leu608Phefs*81) (NM_004655.3). Two variants of uncertain significance were also found (*NF1*, c.6982C > T, p.Arg2328Cys, and *RAD50*, c.1336A > G, p.Lys446Glu). Other genes tested were *ALK, APC, ATM, BAP1, BARD1, BLM, BMPR1A, BRCA1, BRCA2, BRIP1, CASR, CDC73, CDH1, CDK4, CDKN1B, CDKN1C, CDKN2A (p14ARF), CDKN2A (p16INK4a), CEBPA, CHEK2, CTNNA1, DICER1, DIS3L2, EGFR, EPCAM, FH, FLCN, GATA2, GPC3, GREM1, HOXB13, HRAS, KIT, MAX, MEN1, MET, MITF, MLH1, MSH2, MSH3, MSH6, MUTYH, NBN, NF2, NTHL1, PALB2, PDGFRA, PHOX2B, PMS2, POLD1, POLE, POT1, PRKAR1A, PTCH1, PTEN, RAD51C, RAD51D, RB1, RECQL4, RET, RUNX1, SDHAF2, SDHA, SDHB, SDHC, SDHD, SMAD4, SMARCA4, SMARCB1, SMARCE1, STK11, SUFU, TERC, TERT, TMEM127, TP53, TSC1, TSC2, VHL, WRN,* and *WT1.*

The patient completed genetic testing before starting radiation treatment. She was not seen in the Medical Genetics clinic prior to testing, but did watch an educational video on hereditary cancer genetic testing. She was informed of the results by telephone and scheduled into the Medical Genetics Clinic.

**Fig. 3:**
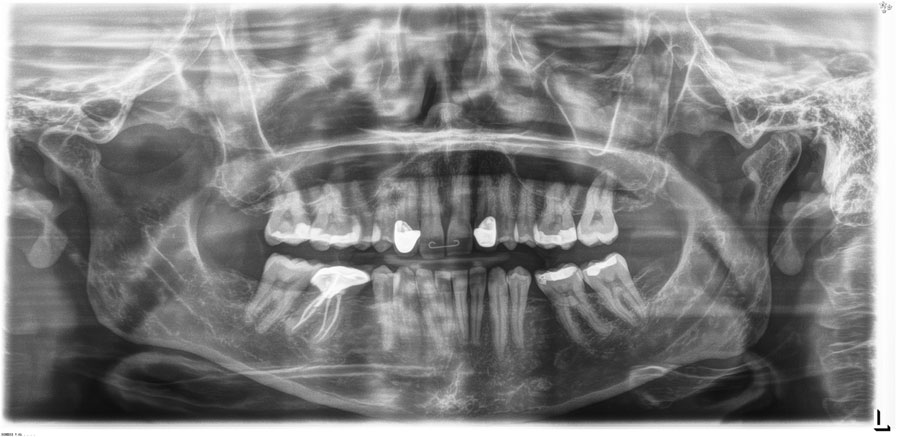
Panoramic Dental Radiograph. Panoramic dental radiograph from our proband with a pathogenic AXIN2 variant. There are 24 teeth. A complete set of adult is 32 teeth. The patient is lacking her upper lateral incisors, bottom second premolars, and 3 third molars (wisdom teeth)

**Fig. 4:**
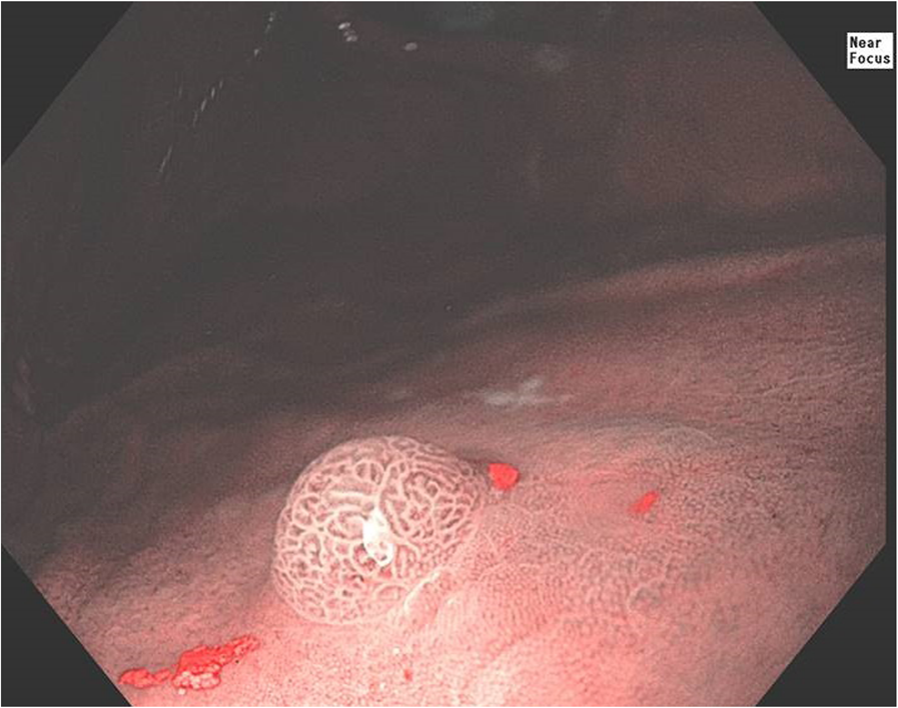
Gastric Adenoma. Endoscopic photograph taken during upper endoscopy from our proband with a pathogenic *AXIN2* variant. Shown is a 4 mm polyp found in the body of the stomach. It was removed with cold forceps and sent to pathology and reported as a gastric adenoma. Photograph taken with narrow band imaging and near focus using an Olympus 190 endoscope

In genetics clinic, she shared history of hypodontia, lacking her upper lateral incisors, bottom second premolars, and 3 wisdom teeth [Fig. 3]. Her upper cuspids had been capped to appear more similar to lateral incisors. On physical examination, eyebrows, hair pattern, and nails were all normal. The patient had no dysmorphic features. The patient had no gastrointestinal symptoms and had never had an upper endoscopy or colonoscopy. Both upper endoscopy and colonoscopy were requested due to association of germline *AXIN2* variants with colorectal neoplasia. In the body of the stomach, there was a small 4 mm polyp [Fig. 4]. It was removed and reported by the pathologist as a gastric adenoma, foveolar type. Both the stomach and duodenum were normal. A 5 mm hyperplastic polyp was removed from the ascending colon.

**Fig. 5:**
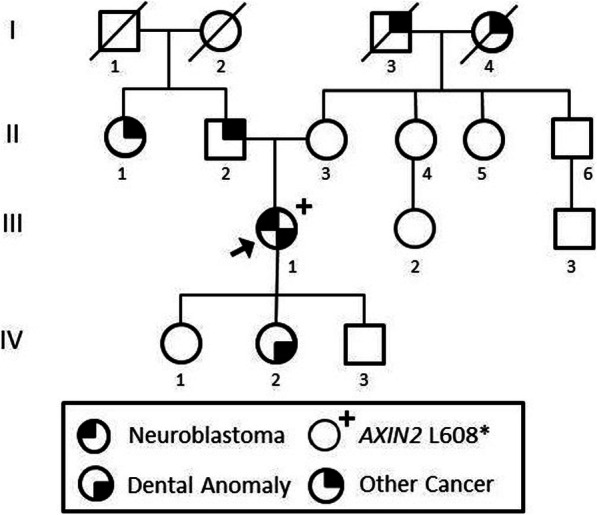
Proband’s Pedigree. Pedigree of our proband carrying a pathogenic *AXIN2* variant. The proband’s father was not available for testing

There was no maternal history of hypodontia or GI polyps [Fig. 5]. Paternal history was incomplete, and no paternal relatives were available for *AXIN2* testing. The patient did recall her father lacked several lower teeth and had a removable lower bridge. Later in life, he was diagnosed with a malignant tumor in his cerebellum, further pathology details unavailable. A paternal aunt had been diagnosed with breast cancer. No further paternal history was available. The proband has three children. Two have typical teeth. The third had a typical number of teeth, but her upper lateral incisors were described as “pegged” and had been capped.

## Discussion and conclusion

We propose that gastric adenomas and olfactory neuroblastoma could be features of the *AXIN2* phenotype. As the total number of patients reported to have pathogenic variants in *AXIN2* is small, this hypothesis is difficult to prove from the current case report population. However, there are other pieces of evidence that support a relationship between germline pathogenic variants in *AXIN2* and gastric adenomas and olfactory neuroblastomas. Gastric adenomas are an established feature of the other Wnt/beta-catenin disorder, familial adenomatous polyposis (FAP). Gastric adenomas are seen in about 10% of those with FAP, and are very uncommon in the general population (< 0.01%) [5]. The subtype of gastric adenoma found in the patient, foveolar, would be even more uncommon in the general population [6].

Neuroblastomas, both olfactory and non-olfactory, are not associated with the FAP phenotype as gastric adenomas are, but other lines support a causative association with germline pathogenic *AXIN2* variants. The proband had negative germline testing for other genes associated with neuroblastoma (*BARD1* and *CHEK2*). *AXIN2* pathogenic variants and olfactory neuroblastoma are both so uncommon that further evaluation of a potential relationship is warranted. Only 311 cases of olfactory neuroblastoma were reported in the Surveillance, Epidemiology, and End Results (SEER) Tumor Registry between 1973 and 2002 [7].

Dysregulation of the Wnt/beta-catenin pathway has been reported in neuroblastoma [8, 9]. The zebra fish *APC* mutant animal model has been reported, in abstract form, to have high rate of esthesioneuroblastoma [10]. Olfactory neuroblastoma have not been reported in the well-studied *APC* (Min1+) mouse model.

Data on germline *AXIN2* variants in patients with olfactory neuroblastomas is very limited. We were unable to find a reported series of patients with olfactory neuroblastoma and clinical *AXIN2* germline genetic testing. Four molecular profiling studies of olfactory neuroblastomas have been published. Three used gene panels not including *AXIN2* [11–13]. One used whole exome sequencing and other technologies on 14 samples [14]. There was no comment on *AXIN2* mutations, however, somatic deletions of either the DMD or LAMA2 loci were present 13 of the 14 samples [Gallia]. In a review paper on genetic patterns in olfactory neuroblastoma, no partial chromosomal deletions were reported involving 17q24.1 [15].

Given the very limited data on *AXIN2* and olfactory neuroblastomas, data from related tumors that are better studied was reviewed. Neuroblastomas originate from sympathetic nerve cells, and olfactory neuroblastomas from olfactory sensory cells. Both the sympathetic nerve cells and olfactory sensory cells are derived from the neural crest.

Germline *AXIN2* variants in patients with neuroblastoma have been previously reported [16] [Table 1]. Three of 52 patients with neuroblastoma studied with whole exome sequencing had *AXIN2* variants. There were no variants in 1000 controls. One of these changes was a non- frameshift deletion, and the three others were nonsynonymous single nucleotide variants. Other than the diagnosis of neuroblastoma, only age of diagnosis and location of neuroblastoma was reported. So it is unknown if any of these four cases had features or family history of *AXIN2-* related disease such as hypodontia.

**Table 1:** Reported Germline *AXIN2* Variants in Patients with Neuroblastoma

Publication	N	Diagnosis Age	Location	Variation	cDNA change	Protein change	Exon	ExAC	ClinVar	SIFT	PolyPhen2
Lasorsa^ab^	1	22 months	Adrenal	missense	c.684G > C	p.L228F	2	Absent	Absent	deleterious	damaging
	2	10 months	Adrenal	non-frameshift deletion	c.1144_1149del	p.382_383del	5	Absent	Absent	–	–
	3	24 months	Not specified	missense	c.1151A > G	p.E384G	5	Absent	Variant of Uncertain Significance	tolerated	benign
				missense	c.1878 T > G	p.S626R	7	8.26e-06	Absent	deleterious	benign
This publication -Macklin^c^	4	49 years	olfactory	frameshift deletion	c.1822del	p.L608*	7	Absent	pathogenic	–	–

^a^Lasorsa VA, et al. Exome and deep sequencing of clinically aggressive neuroblastoma reveal somatic mutations that affect key pathways involved in cancer progression. Oncotarget. 2016 Apr 19;7 (16):21840–52. ^b^NM_004655. ^c^NM_004655.3

Based on our experience and review of the literature, we offer this tentative clinical guidance for the management of pathogenic *AXIN2* carriers:

- Upper endoscopy and colonoscopy beginning at 18 years of age. If there are concerning symptoms, such as rectal bleeding, colonoscopy should be done earlier. Subsequent upper endoscopy and colonoscopy should be done at least every 3 years. If polyps are found, then procedures may need to be done more frequently than every 3 years.
- Yearly history and physical assessment. If patients have any nasal or facial symptoms, we recommend referral to an ear, nose, and throat specialist and imaging.

The small number of known cases with germline *AXIN2* variants makes it challenging to accurately assess risks for these individuals. We would ask that any clinicians treating patients with *AXIN2* pathogenic variants contact us. Our group, working with genetic testing laboratories and other clinical groups, is building an *AXIN2* patient registry.

## Data Availability

The raw datasets generated and/or analyzed during the current study are not publicly available in order to protect participant confidentiality. NM_004655.3 can be accessed through the National Center for Biotechnology Information at https://www.ncbi.nlm.nih.gov/nuccore/NM_004655.3 (accessed 7/29/2020).

## Abbreviations

GI: Gastrointestinal
MRI: Magnetic resonance imaging
FAP: Familial adenomatous polyposis
SEER: Surveillance, epidemiology, and end results
